# Tensile Fatigue Properties of Ordinary Plain Concrete and Reinforced Concrete under Flexural Loading

**DOI:** 10.3390/ma16196447

**Published:** 2023-09-28

**Authors:** Huating Chen, Zhenyu Sun, Xianwei Zhang, Jinhong Fan

**Affiliations:** 1 Faculty of Architecture, Civil and Transportation Engineering, Beijing University of Technology, Beijing 100124, China; szyszy990509@emails.bjut.edu.cn; 2 CCCC First Harbor Engineering Survey and Design Institute Co., Ltd., Tianjin 300222, China; zhangxianwei7@ccccltd.cn; 3 Faculty of Materials and Manufacturing, Beijing University of Technology, Beijing 100124, China; fanjinhong@bjut.edu.cn

**Keywords:** flexural fatigue behavior, ordinary concrete, fatigue *S*-*N* equations, fatigue strain, fatigue stress–strain curve, Weibull distribution, effect of reinforcement

## Abstract

Many bridge structural components are subjected to repetitive vehicle load and temperature gradient action. The resulting cyclic tensile stresses within the structures could cause premature fatigue failure of concrete, dramatically impairing structural components’ durability and sustainability. Although substantial knowledge of fatigue properties on low-strength pavement concrete and high-strength structural concrete has been obtained, research on the most widely used normal-grade ordinary concrete in bridge engineering is still ongoing. Therefore, a four-point bending fatigue test of 97 C50 concrete specimens under a constant amplitude sinusoidal wave was conducted in the laboratory, the flexural fatigue behavior of plain and reinforced concrete specimens was studied, and the cyclic deformation evolution of concrete under fatigue loading was obtained. The empirical fatigue *S*-*N* equations of concrete with a failure probability *p* of 0.1~0.5 were derived through statistical analysis of the test results. The fatigue life of the tested specimens exhibited a two-parameter Weibull distribution. In addition to the maximum stress level *S*_max_, the stress ratio *R* is also a key factor affecting the flexural fatigue life of concrete *N*. The semi-logarithmic and logarithmic equations were almost identical at the tested stress levels, the latter predicting longer fatigue life for *S*_max_ < 0.70. The restraining effect from steel reinforcement slightly lengthened the concrete’s fatigue cracking initiation life. The insight into concrete flexural fatigue properties from this study not only contributes to a better understanding of structural concrete, but also provides a basis for the practical evaluation of concrete or composite bridge decks.

## 1. Introduction

With the increasing vehicle axle loads, concrete bridge decks and other bridge components are prone to fatigue cracking under repeated wheel loading [1]. Once the concrete cracks, the long-term durability of concrete and internal steel reinforcement will be significantly affected, and the service life of the bridge deck will be reduced. Afterward, the elevated tensile stress amplitude induced in the reinforcement and the compressive stress in concrete may lead to performance deterioration or even premature fatigue failure of the bridge deck at loads far lower than its static bearing capacity. As concrete failure is closely related to cracks caused by tension [2], flexural tensile fatigue properties of concrete are fundamental to understanding and evaluating the fatigue cracking behavior of such bridge components, particularly when strengthening existing concrete bridges is necessary.

The flexural tensile fatigue properties of concrete are primarily obtained through constant amplitude fatigue tests of small-scale beam specimens loaded in four-point or three-point bending. Scholars have conducted numerous experimental studies on the flexural tensile fatigue of concrete since the 1970s [3], and the effects of many relevant parameters, such as the stress level, stress ratio, failure probability, and loading frequency, on fatigue life have been investigated [4,5,6]. After statistical and regression analysis of fatigue life data, various forms, semi-logarithm or logarithm, of fatigue life equations have been obtained [5]. For example, concrete bending fatigue *S*-*N* equations under a fixed stress ratio were proposed in the literature [7,8], and probabilistic fatigue *S*-*N* equations considering the influence of the stress ratio and failure probability were proposed in [9,10]. In the recent two decades, the primary focus of research on flexural tensile fatigue properties has shifted towards the effects of fiber reinforcement [11,12], mineral admixtures [13], lightweight aggregates [14,15], and recycled aggregates [16,17,18]. Existing works are mainly conducted on low-strength or high-strength ordinary concrete traditionally and on innovative composite concrete recently. Nevertheless, the research on normal-grade ordinary concrete commonly used in bridge engineering, say, C40~C60, is limited.

While fatigue life is still the primary focus in concrete fatigue research, more and more researchers are paying attention to the accumulation and evolution of fatigue damage during the entire process of fatigue loading [19]. Different physical parameters, such as maximum strain [20], residual strain [21,22], and deformation modulus [23], have been correlated with fatigue damage variables. Experimental investigations that report concrete strain, in addition to applied stress, could provide a fundamental database to study fatigue damage variables and their evolution.

Although reinforced concrete members are often used in actual engineering structures, plain concrete specimens are usually employed in bending fatigue tests. The effect of steel reinforcement on concrete fatigue cracking has yet to be fully explored [24]. Therefore, based on C50 ordinary concrete, which is most widely used in bridge engineering in China, this paper directly compares the difference in fatigue cracking performance between plain concrete and reinforced concrete specimens through four-point bending fatigue tests. The distribution of concrete fatigue cracking life is studied through regression analysis of test data, and the probabilistic fatigue equation of concrete in flexural tension considering the stress ratio and failure probability is obtained. In addition, the longitudinal concrete strain will be recorded during the fatigue test to provide essential data for constructing a concrete fatigue damage constitutive model. With continuously recorded strain data, the novelty of this study lies in obtaining the fatigue characteristics of C50 plain and reinforced concrete specimens through direct comparison.

## 2. Materials and Methods

### 2.1. Materials and Mix Design

The ordinary concrete selected for this study was commercial C50 concrete, thanks to its wide application in bridge engineering. The mix ratio was cement/sand/stone/water = 1:1.93:3.02:0.46. Among these, the cement was ordinary 425 Portland cement produced by a local plant, the sand was medium natural sand with a fineness modulus of 2.4, and the coarse aggregate was rubble and cobble gravel with a maximum particle size of 25 mm. The target slump was 180 ± 20 mm. A high-performance water-reducing agent STD-PCS of 1.99% was added for improved workability. The water-reducing agent STD-PCS was a polycarboxylic acid-type superplasticizer manufactured by a local company (Tianjin Steady Industrial Development Co., Ltd., Tianjin, China). The workability of fresh mixtures was evaluated with the standard slump cone with dimensions of a 100 mm diameter on the top, a 200 mm diameter on the bottom, and 300 mm high. More information about the concrete mix is given in Table 1.

The steel reinforcement under consideration was the commonly used grade HRB400. The standard values of the steel’s yield strength, tensile strength, elastic modulus, and elongation percentage are 400 MPa, 540 MPa, 200 GPa, and 16%, respectively.

### 2.2. Specimen Preparation

**Figure 1:**
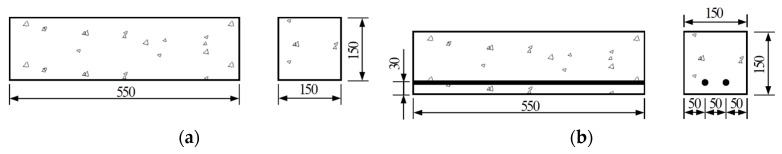
Beam specimens (dimensions in mm): (**a**) plain concrete specimens in PC and RC series; (**b**) reinforced concrete specimens in RC series.

Standard flexural strength specimens with dimensions of 150 mm × 150 mm × 550 mm, as shown in Figure 1 and specified in the Chinese Standard GB/T 50081-2019 for the test method of mechanical properties on ordinary concrete [25], were adopted. Altogether, 132 specimens were prepared in two series, 66 specimens for each, namely a PC series and RC series. All PC specimens were made from plain concrete, while the RC series consisted of 48 reinforced concrete beams supplemented by 18 plain concrete beams. Six companion 150 mm × 150 mm × 150 mm concrete cubes were obtained for each beam specimen series to verify the concrete grade through the compressive strength test.

The reinforced concrete specimens were identical to their plain concrete counterparts except for reinforcement. A reinforcement ratio of 1%, which is typical in concrete bridge decks, was provided in the reinforced concrete specimens. Two full-length grade HRB400 Φ12 mm reinforcing bars were placed in the bottom part of each specimen, with a concrete cover of 30 mm. A schematic diagram of the reinforced concrete specimens is shown in Figure 1b.

**Figure 2:**
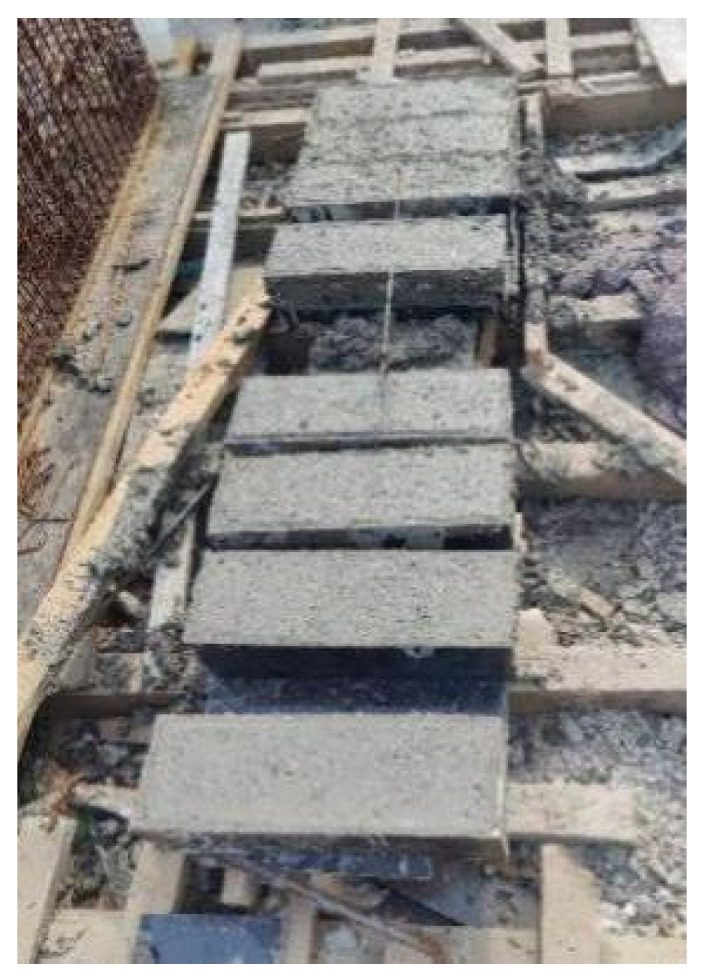
Specimen preparation during concrete pouring.

All specimens were produced with a wooden formwork. After concrete pouring, the specimens were covered with a polyethylene sheet and cured for 28 days under standard curing conditions (at a temperature of 22 °C and a relative humidity of 95%). After 28 days of standard curing, the specimens were stored in the Mechanics Laboratory near the testing machine. No special treatment was provided. Roughly, they were exposed to a room temperature of 17~27 °C and relative humidity of 30~80%. At the time of testing, the age of the specimens was 35~60 days. Figure 2 shows a photo of the specimen preparation during the concrete pouring.

### 2.3. Testing Set-Up and Loading Matrix

**Figure 3:**
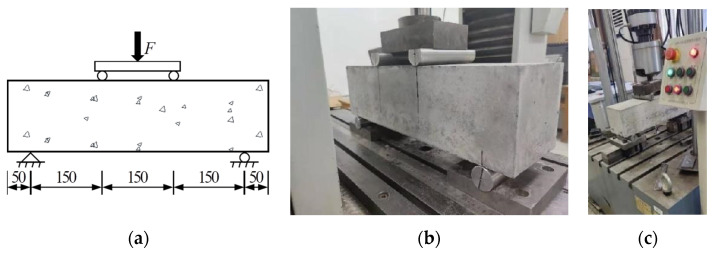
Four-point bending test: (**a**) scheme of test set-up (dimensions in mm); (**b**) photo of static test set-up; (**c**) photo of fatigue test set-up.

All beam specimens were tested under four-point bending conditions, as shown schematically in Figure 3a. A standard static flexural test was conducted before the fatigue test to obtain the flexural strength. A total of 9 specimens from the PC series and 24 specimens (including 18 plain concrete beams and 6 additional reinforced concrete beams) from the RC series were tested by a static test machine, shown in Figure 3b. For the reinforced concrete specimens, the flexural strength was determined when a macroscopic concrete crack appeared at the side surfaces of the tested beam.

The fatigue test was conducted with a QBS-50A electro-hydraulic servo-controlled universal testing machine in the Engineering Mechanics Laboratory at the Beijing University of Technology. The testing machine, manufactured by Changchun Qianbang Testing Equipment Co., Ltd. (Changchun, China), has a maximum load capacity of 50 kN and a loading frequency of 0.1~10 Hz, as shown in Figure 3c.

A fatigue loading matrix with combinations of the maximum and minimum stress levels was designed. Stress levels are defined as the fatigue stresses divided by the static strength. The stress ratio *R* is the ratio between the minimum stress level *S*_min_ and the maximum stress level *S*_max_. *S*_max_ in the range of 0.65~0.90, and *S*_min_ of 0.10, 0.25, and 0.4 were considered in the test program. The fatigue test matrix for the PC series (plain concrete specimens) and RC series (reinforced concrete specimens) is shown in Table 2. A constant amplitude sinusoidal wave was applied to maintain the desired stress levels. Due to the limitation of the testing machine, 15 specimens with a maximum stress level of 0.9 were tested with a slower frequency of 0.1 Hz. All other specimens were tested with a frequency of 5 Hz. Specimens were positioned correctly on the fatigue testing machine, and the parameters were adjusted to obtain the desired fatigue load waveform and frequency, as shown in Figure 3c.

Fatigue failure is defined when a plain concrete specimen breaks or a macroscopic concrete crack appears at the side surfaces of a reinforced concrete specimen. The fatigue test was terminated at the time of failure or after a predetermined number of cycles. A specimen undergoing no signs of failure after 1 million loading cycles was terminated in the fatigue test for plain concrete specimens and was termed run-out. For the reinforced concrete specimens, fatigue loading usually continued after the first sign of concrete cracking until far surpassing the observed fatigue life of the plain concrete counterparts tested under similar stress levels. The predetermined number of cycles for the fatigue test termination of the RC series was between 10,000 and 80,000, depending on the tested stress levels.

### 2.4. Measurement and Instrumentation

**Figure 4:**
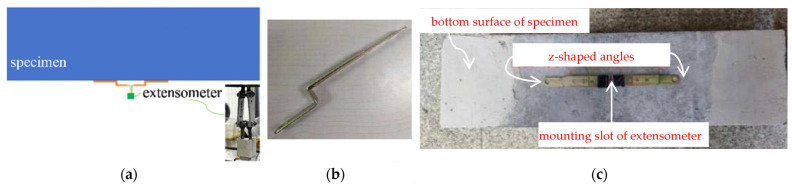
Devices to extend the range of extensometer measurements: (**a**) scheme of strain measurement; (**b**) Z-shaped angle; (**c**) arrangement of angles for extensometer installation.

The fatigue testing machine automatically recorded load, displacement, and the number of cycles of the actuator, and a real-time stress-versus-time curve was displayed to facilitate test monitoring. For continuous measuring of the strain during the fatigue test, an extensometer was used to determine the average strain at the bottom surface of the specimen over the pure bending region, i.e., the middle third of the span. A YYJ-(–2)-5/6 extensometer, manufactured by NCS Testing Technology Co., Ltd. (Beijing, China), has a default gauge length of 6 mm, a measurement range between –2 mm and 5 mm, and a measuring sensitivity of 0.001 mm. Rigid Z-shaped angles were glued to the bottom surfaces of test specimens to ensure cracks occurred within the gauge length. The gauge length was thus extended to 90 mm for plain concrete specimens and 146 mm for reinforced concrete specimens, respectively. Figure 4 shows the arrangement of Z-shaped angles.

### 2.5. Fatigue Life Distribution

The observed fatigue life of concrete specimens is a random variable with great discreteness. When the fatigue life data were arranged in ascending order, the failure probability *p* of a particular specimen could be estimated by the average rank as in the following expression [14]:

(1) $$p=\frac{i}{k+1}$$

where *i* denotes the failure order, and *k* is the total number of specimens tested at a particular stress level.

Concrete fatigue life is generally assumed to conform to the two-parameter Weibull distribution [4,9]. Accordingly, the failure probability *p* satisfies Equation (2). Equation (3) can be derived to test whether a group of experimental results follows the two-parameter Weibull distribution. Suppose the regression analysis of the experimental data can show a good statistical linear relationship between lnln(1/(1 − *p*)) and ln*N*; that is, the coefficient of determination *R*^2^ is relatively high. In that case, this assumption is valid, and vice versa.

(2) $$p=F(N)=1-e^{-N^{m}/t_{0}}\;\;\;(N\geq 1\;and\;t_{0}>0)$$

(3) $$lnln(1/(1-p))=mlnN-lnt_{0}$$

where *m* is the shape parameter, and the scale parameter can be expressed as ${t_{0}}^{1/m}$.

Recall that the experiment program includes specimens tested with combinations of the maximum and minimum stress levels, as described in Section 2.3 and Table 2. While the maximum stress level *S*_max_ is of primary concern, it is desirable to consider the effect of the stress ratio *R* on the probabilistic distribution of fatigue life. According to the literature [9], using equivalent fatigue life, that is, $\bar{N}=N^{1-R}$, all experimental data with the same *S*_max_ can be combined for statistical analysis. The equations for the Weibull distribution test can be rewritten as follows:

(4) $$p=F(\bar{N})=1-e^{-{\bar{N}}^{m}/t_{0}}\;\;\;(\bar{N}\geq 1\;and\;t_{0}>0)$$

(5) $$lnln(1/(1-p))=mln\bar{N}-lnt_{0}$$

### 2.6. S-N Curves

Various fatigue *S*-*N* equations have been proposed. The maximum stress level *S*_max_ has traditionally been written as *S* for conciseness. While the dependent variable fatigue life *N* is always expressed in logarithm, the independent variable can either be *S* or the logarithm of *S*. Some *S*-*N* curves apply to fatigue tests conducted at a fixed minimum stress level, usually close to 0, and others can consider the effect of the stress ratio *R*.

The semi-logarithmic and logarithmic fatigue equations for specimens fatigue-tested with a fixed minimum stress level are expressed as follows:

(6) S=a−b lgN

(7) lgS=A−B lgN

where intercept *a* or *A* is the basic strength corresponding to *N* = 1, and slope *b* or *B* is called the fatigue strength exponent.

The semi-logarithmic and logarithmic fatigue equations, when considering the stress ratio *R*, can be expressed as follows:

(8) $$S=a-b(1-R)\;lgN=a-b\;lg\bar{N}$$

(9) $$lgS=A-B(1-R)\;lgN=A-B\;lg\bar{N}$$

where the constants *a*, *b*, *A*, and *B* have the same physical meaning as those in Equations (6) and (7), except equivalent fatigue life $\bar{N}$ is considered.

A direct quantitative relationship between fatigue life and failure probability could be established for reliability analysis applications. Probabilistic fatigue equations are obtained by substituting the fatigue life of various failure probabilities into Equations (6)–(9), Equations (6) and (7) for constant *S*_min_ of 0.10, and Equations (8) and (9) when considering the stress ratio. If the fatigue life or equivalent fatigue life correlates with two-parameter Weibull distribution, the fatigue life *N*, or equivalent fatigue life $\bar{N}$ when considering the stress ratio, corresponding to a given failure probability *p*, can be calculated by Equation (10) by rewriting Equations (2) and (4).

(10) $$N\;or\;\bar{N}={\left[ln(1/(1-p))\times t_{0}\right]}^{1/m}$$

## 3. Results and Discussion

Fatigue test results were obtained from 55 plain concrete specimens (PC series) and 42 reinforced concrete specimens (RC series). The maximum and minimum fatigue stresses were determined from the preset stress levels (Table 2) and the average flexural strength of each series. The compressive strength of concrete cubes was utilized to verify the strength grade of the concrete mixes.

### 3.1. Test Results

#### 3.1.1. Material Characterization

The compressive strength was obtained from standard tests on six cubic specimens for each series. The test results, along with the statistical values, are shown in Table 3. The 28-day average compressive strengths of the PC and RC series were 53.9 MPa and 51.1 MPa, respectively. Both concrete batches met the strength requirement of commercial grade 50 concrete, while the RC series had a 5% lower average strength than the PC series. However, the material dispersion of the RC series was significantly smaller than that of the PC series.

Concrete flexural strength was obtained from standard four-point bending tests on 9 PC series beam specimens and 24 RC series specimens. The six beams in Sets 7 and 8 of the RC series were specimens with reinforcement. While all plain concrete specimens fractured in the middle third region, the reinforced concrete specimens did not fail due to the strength of reinforcement. Therefore, the flexural strength of the concrete in the reinforced concrete specimens was determined when macroscopic cracks appeared on the side surfaces of the beams.

The measured static flexural strengths of the two batches of concrete, along with the statistical values, are shown in Table 4. The average flexural strength of the RC series was 9% lower than the PC series, which is consistent with the relative values of their compressive strength. The coefficients of variation between the two series are almost identical. Statistically, reinforced concrete specimens show slightly smaller flexural strength than plain concrete specimens.

#### 3.1.2. Failure Mode

**Figure 5:**
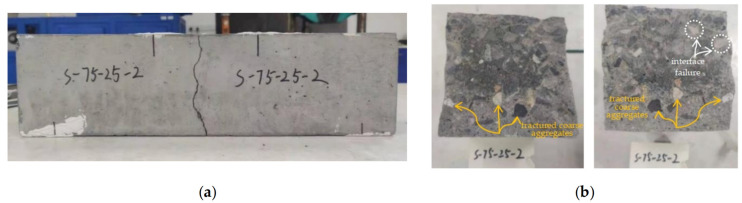
Typical fatigue failure of plain concrete specimens (PC series): (**a**) elevation view; (**b**) cross-section view.

Towards the end of the fatigue test, a vertical crack was observed to develop in the middle region in most plain concrete specimens, followed by a sudden fracture. The location of the fracture surface was random. However, all beams failed in the middle third pure bending region, and the fracture surface was generally planar. Most of the coarse aggregates were fractured, and a few pullouts of coarse aggregates along the interface between the coarse aggregates and cement mortar made the fracture surface zigzag. A typical fatigue failure of the plain concrete specimen is presented in Figure 5.

**Figure 6:**
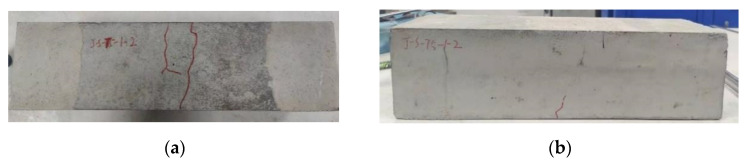
Typical fatigue failure of reinforced concrete specimens (RC series): (**a**) bottom surface; (**b**) side surface.

For reinforced concrete specimens under fatigue loading, small cracks also occurred initially on the bottom surface within the pure bending region. After these cracks became macroscopic, they appeared on the side surfaces of the concrete specimen and slowly extended upward with the application of cyclic loading. At the same time, concrete cracks continued to develop at other locations of the pure bending region and gradually merged. Since there was tensile reinforcement, the specimen stiffness did not change apparently after concrete cracking, and it did not break abruptly. As the fatigue cracking life of concrete was the primary interest in this work, the fatigue test was stopped after visible cracks appeared on the side surfaces of specimens. A typical concrete fatigue failure of the reinforced concrete specimen is shown in Figure 6.

#### 3.1.3. Fatigue Life

Fatigue life is the number of cycles a specimen can sustain before failure. The fatigue life of plain concrete specimens is summarized in Table 5. The specimen number is designated as S-a-b-x, where S means the tests are under stress control, a stands for the maximum stress level, b represents the minimum stress level, and x is the order of testing within the test set.

Even in carefully controlled tests, it is observed in Table 5 that a significant difference in the fatigue life of several orders of magnitude existed for specimens within the same test set. The fatigue life results between different test sets are also remarkably different. Generally, the fatigue life increased as the maximum stress level decreased when the minimum stress was kept constant or as the minimum stress level increased under the same maximum stress. Both the maximum and minimum stress levels affected the concrete’s fatigue life.

Similarly, the reinforced concrete specimen is designated with an additional “J”, as J-S-a-b-x, where all other parameters have the same meaning as in the PC series. Table 6 presents the fatigue cracking life data of the RC series in ascending order.

#### 3.1.4. Strain Evolution

With an extensometer installed on the bottom fiber of the specimen, the longitudinal deformation and strain of the concrete were continuously recorded during the fatigue test. Under fatigue loading, the concrete strain increased non-uniformly with the elapse of loading cycles. This phenomenon was observed in all specimens under different stress levels.

**Figure 7:**
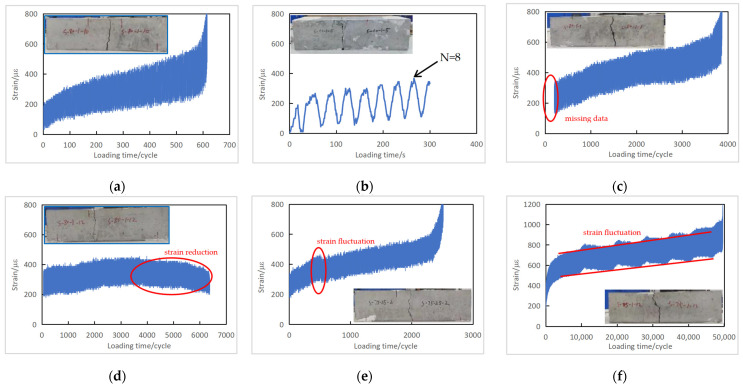
Fatigue strain versus loading time curves of plain concrete specimens (PC series): (**a**) Specimen S-80-1-10; (**b**) Specimen S-90-1-5; (**c**) Specimen S-80-1-1; (**d**) Specimen S-80-1-12; (**e**) Specimen S-75-25-2; (**f**) Specimen S-75-1-12.

Figure 7 lists the test results from the PC series, including the measured strain-versus-time curves and photos of the failed specimens.

The strain-versus-time curve of a typical plain concrete specimen in Figure 7a can be divided into three stages: the first stage, where the concrete strain increases rapidly at the beginning of loading; the second stage, during which the strain tends to be stable and grows slowly; and the third stage, when approaching the end of loading, where the strain increases rapidly, and the specimen breaks suddenly. The second stage accounts for most of the fatigue life, approximately 80% on average.

The strain amplitude is the difference between the corresponding strains under the maximum and the minimum loads. While the strain amplitude stays stable for most specimens during the fatigue loading cycles, the increase in the strain amplitude is prominent for specimens tested under higher stress levels and whose fatigue life is less than 1000 cycles, as shown in Figure 7a,b. In general, concrete deforms in three stages during the fatigue loading of most PC series specimens, and the smaller the maximum stress level, the more distinctive the three-stage feature. These deformation characteristics are consistent with the three-stage rule of concrete fatigue flexural failure recorded in the literature [7].

Some peculiarities in strain measurement were observed during the fatigue testing. For example, in Figure 7c, the initial rapid strain growth stage was missed in Specimen S-80-1-1 because the first 200 cycles were not recorded due to an operational mistake. Some specimens were recorded with strain–time curves similar to Figure 7d, where a strain reduction occurred after some fatigue cycles. The corresponding failure photos show that in these specimens, concrete failure occurred outside the measuring range of the extensometer. The range of the extensometer for the PC series was extended to 90 mm. However, the pure bending region was 150 mm (the gauge length was subsequently extended to 146 mm for the RC series). When a specimen cracked outside the measuring range of the extensometer, the corresponding concrete deformation was more significant than that within the measuring range, resulting in the concrete being squeezed and the measured strain decreasing with the increase in loading cycles. Figure 7e,f, for Specimens S-75-25-2 and S-75-1-12, shows strain fluctuation. Because the fatigue test duration was relatively long for specimens with lower stress levels, the machine vibration and other unintentional disturbances may have affected the strain extensometer recording.

For the test specimens whose fracture occurred within the measurement range of the extensometer, the measured minimum and maximum strains during the last cycle of fatigue loading are summarized in Table 7, where the influence of machine vibration on the extensometer measurement has been eliminated. The residual strain, the remaining strain when the load is reduced to 0, was linearly extrapolated from the measured minimum and maximum strains and the applied stresses. The deformation modulus, the slope of the line connecting the loading and unloading tips, of the last fatigue cycle, is also reported.

As seen in Table 7, for specimens with a minimum stress level of 0.10, the maximum strain in the plain concrete specimen during the last fatigue cycle increased from 578 με to 1164 με as the maximum stress level decreased from 0.90 to 0.75. For specimens tested under the same maximum stress level, the minimum strain and the corresponding residual strain at the last fatigue cycle increased as the minimum stress level increased from 0.10 to 0.25, implying more significant fatigue damage with a lower stress ratio. However, the coefficient of variation of the maximum strain, minimum strain, residual strain, and the deformation modulus at failure was large, indicating significant discreteness in the fatigue material properties. This large dispersion may be caused by the discreteness of the concrete material and its static strength [26]. Moreover, measurement error may have influenced the minimum and maximum strains at fatigue failure.

The number of cycles when a macroscopic crack was observed on the side surfaces of a reinforced concrete specimen is shown in the figure as a dotted line, and the right end shows when the fatigue test was terminated.

**Figure 8:**
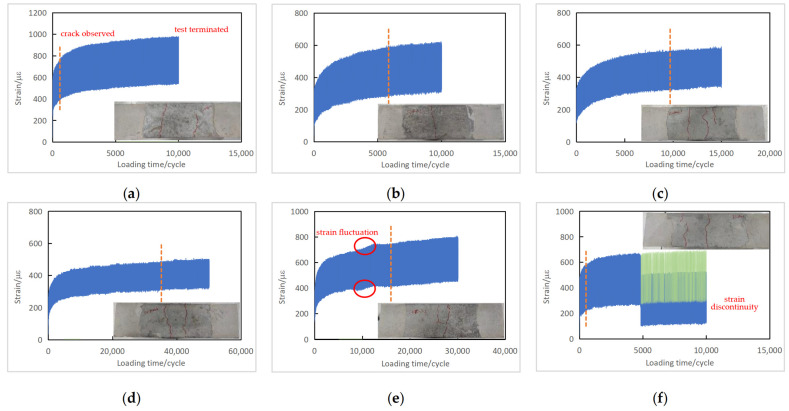
Fatigue strain versus loading time curves of reinforced concrete specimens (RC series): (**a**) Specimen J-S-85-1-5; (**b**) Specimen J-S-80-1-5; (**c**) Specimen J-S-80-25-1; (**d**) Specimen J-S-75-25-3; (**e**) Specimen J-S-75-1-2; (**f**) Specimen J-S-85-1-3.

For the typical reinforced concrete specimens shown in Figure 8a–d, the development of fatigue strain was initially consistent with that of the plain concrete specimens: the concrete deformed rapidly at the first stage of loading and gradually stabilized at the second stage. Unlike the plain concrete specimen, the rapid growth of the concrete strain in the third stage was not observed in the RC series. In contrast to the sudden fracture in the PC series once the concrete cracked, concrete cracking in the RC series did not lead to brittle fracture of the beam specimen. This ductile behavior is the consequence of the reinforcement effect. When concrete starts cracking, the tensile stress in the bottom part is sustained by the reinforcement; therefore, concrete strain tends to be stable, limiting the upward propagation of concrete cracks. By the time the fatigue test was terminated beyond the first detection of a macroscopic concrete crack, concrete fatigue strain in the PC series remained in the stable stage of strain development. Also, because of the role of reinforcement after concrete cracking, the strain amplitude in the reinforced concrete specimens did not increase rapidly, even for specimens tested under higher stress levels.

The strain-versus-time curve in Figure 8e shows the strain fluctuation similarly observed in the PC series. Moreover, Figure 8f, for Specimen J-S-85-1-3, shows an apparent strain discontinuity at about 5000 cycles. This peculiarity is due to an accidental touch to the extensometer. The adjusted strain, shown as the green lines in Figure 8f, was obtained by simply translating the recorded strain data.

To analyze the fatigue strain development in the reinforced concrete specimens, the measured minimum strain, measured maximum strain, and extrapolated residual strain (the remaining strain if the specimen is unloaded to 0) just before fatigue concrete cracking of the specimens are summarized in Table 8.

As observed in Table 8, the fatigue strain of the reinforced concrete specimens evolved similarly to the plain concrete specimens. The maximum strain at failure increased with the decrease in the maximum stress level. However, the maximum strain in the reinforced concrete specimens was generally smaller than that in the plain concrete specimens, which may have been caused by the existence of reinforcement. Reinforcement can restrict concrete deformation, resulting in a lower maximum strain and coefficient of variation in the RC series.

#### 3.1.5. Cyclic Stress–Strain Curves

**Figure 9:**
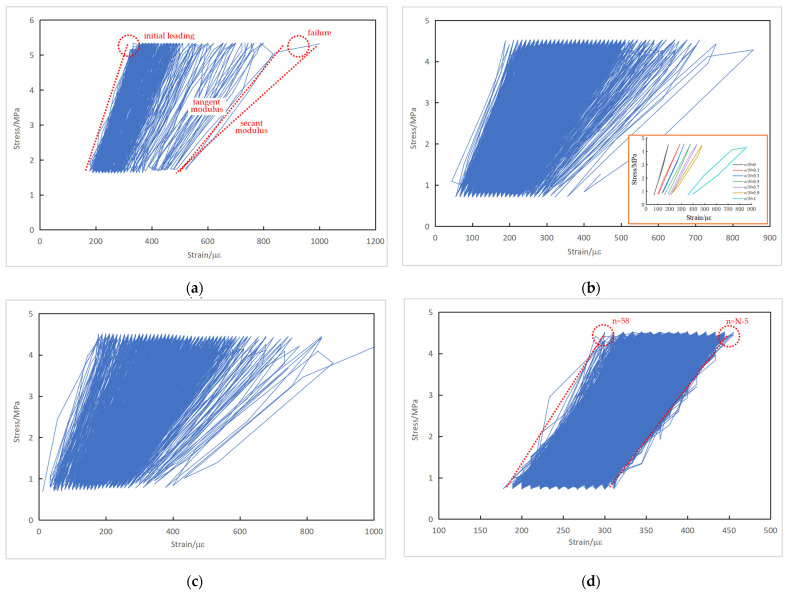
Cyclic stress–strain curves of plain concrete specimens: (**a**) Specimen S-90-25-2; (**b**) Specimen S-80-1-5; (**c**) Specimen S-80-1-10; (**d**) Specimen S-80-1-12.

A continuous cyclic stress-versus-strain curve can be obtained, with stress as the ordinate and strain as the abscissa. The measured cyclic stress–strain curves of typical plain concrete specimens are shown in Figure 9.

Since the fatigue test was under load control, both the maximum and minimum stresses remained constant during fatigue loading. As shown in Section 3.1.4, the strain responses due to fatigue loading kept increasing. The cyclic stress–strain curves in Figure 9 thus demonstrate the evolution of the concrete’s response from the beginning towards the end of the fatigue test. The slope of the cyclic stress–strain curve, termed the deformation modulus of concrete, decreased with the application of fatigue loading. A remarkable decrease in the deformation modulus and differentiation between the tangent and secant modulus was observed before fatigue failure. Figure 9d seems to be an exception, as the strain data of the first 57 and last 5 cycles were not recorded due to operation mistakes. This figure demonstrates that the deformation modulus exhibited a typically three-stage evolution in which the stiffness response was reasonably stable during stage II.

The stress–strain curves at the specified cycle ratio *n*/*N* are also depicted in Figure 9b to illustrate the stress–strain evolution more clearly, where *n* is the number of fatigue loading cycles and *N* is the total fatigue life. During the earlier stages of fatigue loading, the stress–strain response became more linear elastic. With the increase in the cycle ratio, plastic damage in the concrete accumulated gradually. As the fatigue loading continued, the loading and unloading branch in a cycle tended to form a hysteresis loop. The area of the hysteresis loop, to some extent, represents the strain energy consumed by the concrete and the fatigue damage accrued during each fatigue loading cycle.

**Figure 10:**
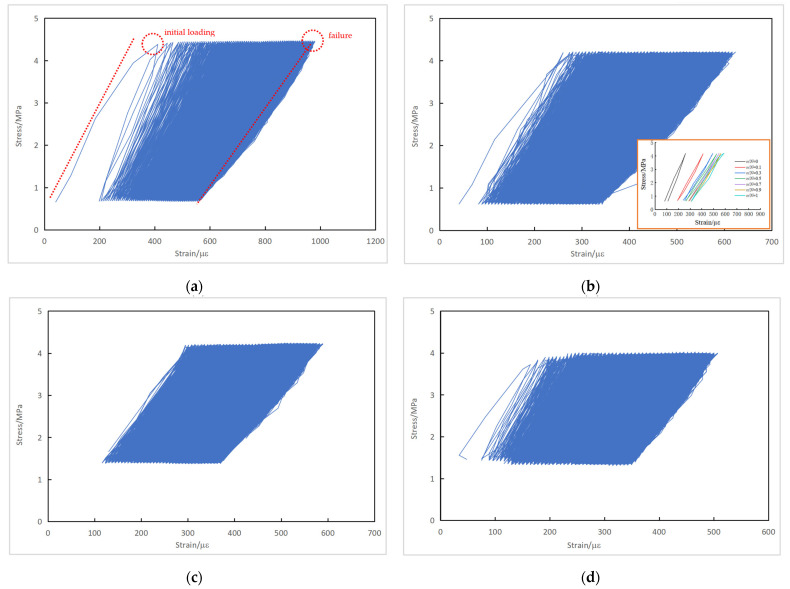
Cyclic stress–strain curves of reinforced concrete specimens: (**a**) Specimen J-S-85-1-5; (**b**) Specimen J-S-80-1-5; (**c**) Specimen J-S-80-25-1; (**d**) Specimen J-S-75-25-3.

The measured cyclic stress–strain curves of typical reinforced concrete specimens are shown in Figure 10.

Similarly, the cyclic stress–strain curves of the reinforced concrete specimens show a decrease in the deformation modulus as the fatigue loading continued. Because of restraint from the reinforcement, the decreasing trend in the concrete deformation modulus and specimen stiffness in the reinforced concrete specimens with the number of loading cycles was less noticeable compared to the plain concrete specimens. While a significant decrease in the deformation modulus within the last 10% of fatigue life for the PC series is evident in Figure 9b, Figure 10b shows that the change in the deformation modulus of the RC series was marginal when *n*/*N* increased from 0.5 to 1.

### 3.2. Probabilistic Analysis of Fatigue S-N Curves

#### 3.2.1. Probabilistic Distribution of Fatigue Life

**Figure 11:**
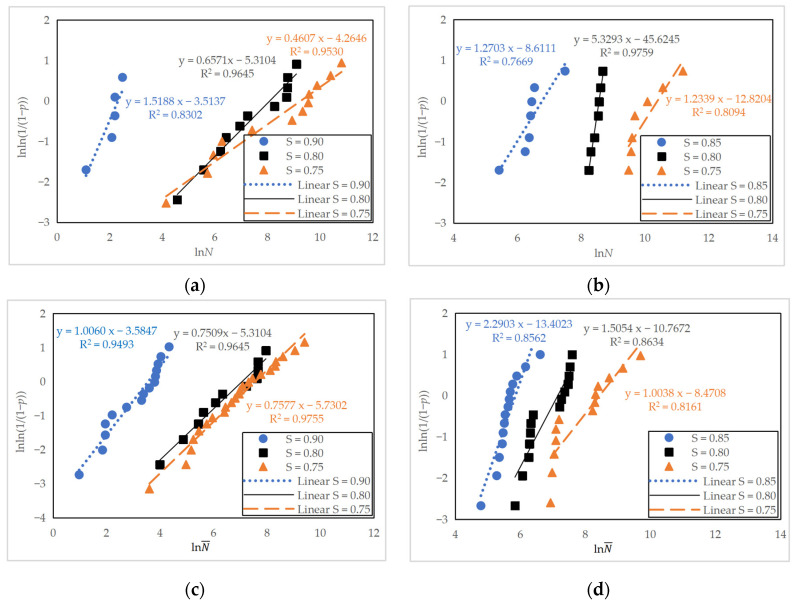
Weibull distribution test of fatigue life: (**a**) Plain concrete specimens (PC series) at a fixed minimum stress level of 0.10; (**b**) reinforced concrete specimens (RC series) at a fixed minimum stress level of 0.10; (**c**) plain concrete specimens (PC series) at varying minimum stress levels; (**d**) reinforced concrete specimens (RC series) at varying minimum stress levels.

In Section 3.1.3, three specimens tested at *S*_max_ of 0.65 did not fail after 1 million cycles and were termed run-outs. Four outliers in Table 5 and Table 6 of the fatigue life data were identified by employing Chauvenet’s criterion [27]. These data points were excluded from further examination. Statistical analysis on two datasets of specimens was performed. One dataset of specimens comprised specimens tested at a fixed minimum stress level of 0.10; the other dataset consisted of specimens tested at varying minimum stress levels, and therefore, the effect of the stress ratio could be considered. Figure 11 shows the Weibull distribution test of the fatigue life of the plain concrete and reinforced concrete specimens, and the corresponding regression analysis results are listed in Table 9. It is worth emphasizing that the equivalent fatigue life was adopted when considering the stress ratio. The slope and intercept terms in the linear equations presented in Figure 11 correspond to the shape parameter *m* and ln*t*_0_ in Equations (3) and (5), respectively.

It can be seen in Figure 11 and Table 9 that the coefficient of determination was generally higher for specimens with varying minimum stress levels where more data points were available. Under each stress level, the coefficient of determination exceeded 0.75, indicating a linear relationship between lnln(1/(1 − *p*)) and ln*N* (or $ln\bar{N}$). Therefore, both the fatigue life and equivalent fatigue life correlate reasonably well with the two-parameter Weibull distribution. A linear regression analysis of the fatigue equations is thus presented in the following sections.

#### 3.2.2. Mean Fatigue *S*-*N* Curves

**Figure 12:**
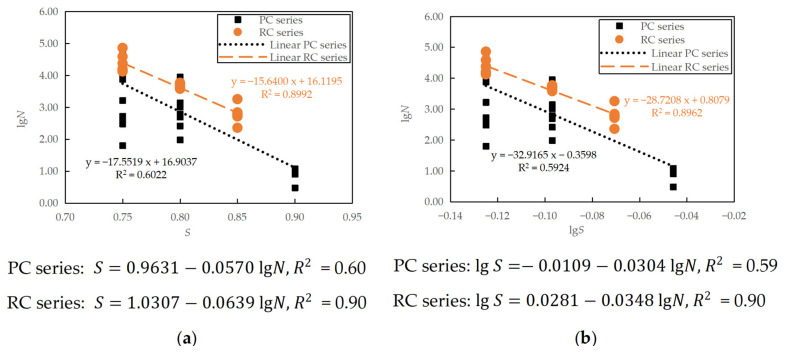
Mean fatigue equations at a fixed minimum stress level of 0.10: (**a**) semi-logarithmic equation; (**b**) logarithmic equation.

When the minimum stress level was fixed as 0.10, the fitting of the plain concrete (PC series) and reinforced concrete (RC series) fatigue test results to the fatigue *S*-*N* curves are shown in Figure 12. Note that the constant terms in the linear equations presented in the figure were obtained from the regression analysis of the fatigue life as dependent variable y and the stress level as independent variable x. The constants *a*, *b*, *A*, and *B* in Equations (6) and (7) were then obtained from simple transformation.

It can be seen in Figure 12 that under the conditions of a fixed minimum stress level, the fatigue equation fitting of the RC series specimens was reasonably good, with the *R*^2^ close to 0.90. However, the *R*^2^ of the PC series was about 0.60, which indicates that only 60% of the variation in *N* can be explained by the regression relationship. Large discreteness can be observed between the PC specimens with the same stress level, particularly at a lower stress level of 0.75. The reinforced concrete specimens show a slightly longer fatigue life than the plain concrete counterparts, indicating that although reinforcement mainly controlled the fatigue crack propagation in the reinforced components, the existence of reinforcement did affect the flexural fatigue cracking of concrete beneficially.

**Figure 13:**
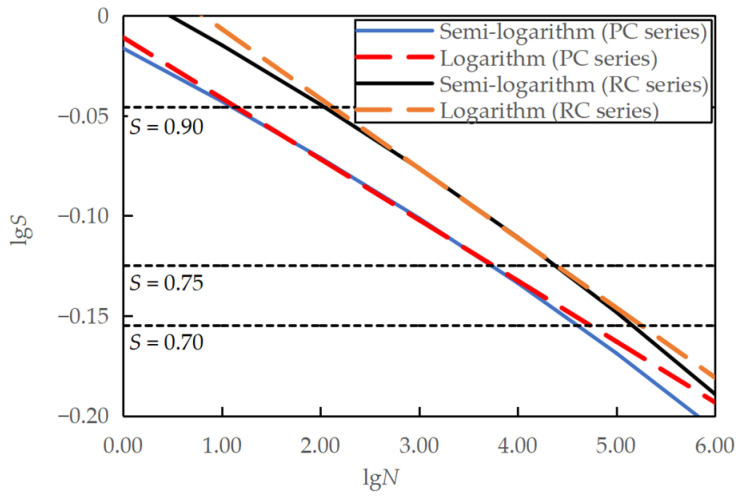
Comparison between semi-logarithmic and logarithmic equations for specimens tested at a fixed minimum stress level of 0.10.

By taking the logarithm of *S* and substituting S with the semi-logarithmic fatigue equation, a relationship between lg*S* and lg*N* can be obtained, that is, lg S=lg (0.9631−0.0570 lgN) for the PC series and lg S=lg (1.0307−0.0639 lgN) for the RC series, which can then be compared with the logarithmic fatigue equations, as shown in Figure 13. The two equations are equivalent to each other for the stress levels studied. However, when it is necessary to extend the stress level *S* to a lower value of less than 0.70, the logarithmic fatigue equation predicts a fatigue life longer than the semi-logarithmic fatigue equation. This observation is consistent with previous research [7].

**Figure 14:**
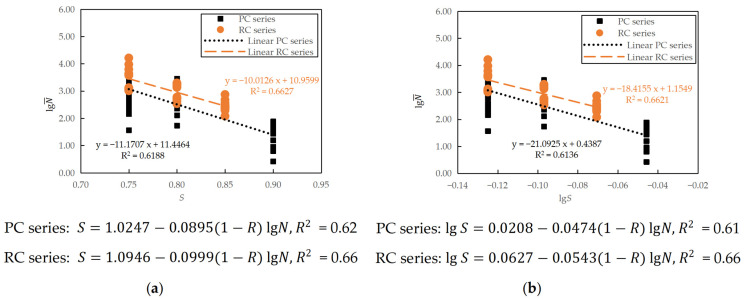
Mean fatigue equations considering the effect of stress ratio: (**a**) semi-logarithmic equation; (**b**) logarithmic equation.

The maximum stress level *S* was also taken as an independent variable when considering the influence of the stress ratio *R*. Similar to Section 3.2.1, the equivalent fatigue life $\bar{N}$, which relates to the stress ratio *R* and fatigue life *N* as $\bar{N}=N^{1-R}$, is used to combine all experimental data at the same maximum stress level [9]. This $\bar{N}$ is then treated as the dependent variable in regression analysis, making fitting linear fatigue equations convenient in practical applications. The regression analysis of semi-logarithmic and logarithmic fatigue equations, considering the effect of the stress ratio, of the plain and reinforced concrete specimens are shown in Figure 14. The figure also shows the corresponding linear regression equations, from which the constants *a*, *b*, *A*, and *B* in Equations (8) and (9) were obtained through simple transformation.

It can be seen in Figure 14 that when considering the stress ratio *R*, the fatigue equations of the plain and reinforced concrete specimens are comparable, with the RC series showing a slightly longer fatigue life. However, the coefficients of determination of the fitted equations are not high (0.61~0.66). This low linearity correlation reflects the complexity of the concrete fatigue phenomenon, especially in the sense of significant variation.

**Figure 15:**
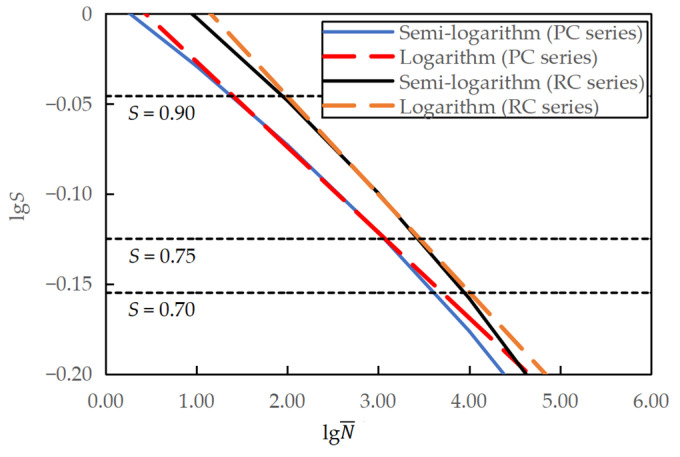
Comparison between semi-logarithmic and logarithmic equations for specimens tested at varying minimum stress levels.

Similarly, by taking the logarithm of *S* and substituting *S* with the semi-logarithmic fatigue equation, a relationship between lg*S* and lg*N* can be obtained, that is, $lg\;S=lg\;(1.0247-0.0895\;lg\bar{N})$ for the PC series and $lg\;S=lg\;(1.0946-0.0999\;lg\bar{N})$ for the RC series, which can then be compared with the logarithmic fatigue equations, as shown in Figure 15. The two equations were identical for the studied stress levels. However, the logarithmic fatigue equation predicted a longer fatigue life than the semi-logarithmic fatigue equation for stress levels smaller than 0.70.

The mean fatigue *S*-*N* curves from this study are summarized in Table 10.

#### 3.2.3. Probabilistic Fatigue *S*-*N* Curves

The regression analysis in Section 3.2.2 obtained fatigue equations corresponding to a failure probability of approximately 50%. By substituting the regression parameters *m* and *t*_0_ from Table 9 into Equation (10), the fatigue life and equivalent fatigue life under different failure probabilities at various stress levels for the plain concrete specimens (PC series) and reinforced concrete specimens (RC series) was obtained and are listed in Table 11.

The data in Table 11 are fitted to semi-logarithmic Equations (6) and (8) and logarithmic Equations (7) and (9), respectively, and the regression coefficients *a*, *b*, *A*, and *B* under different failure probability *p* were obtained. The values are shown in Table 12. The smallest *R*^2^ value of the regression analysis was obtained as 0.83 under a failure probability of 0.1, indicating a linear relationship between *S* (or lg*S* for logarithmic equation) and lg*N*. Comparing Table 10 and Table 12, probabilistic fatigue equations derived in this section with a failure probability of 0.5 are very close to those developed in Section 3.2.2, indicating the validity of probabilistic analysis.

**Figure 16:**
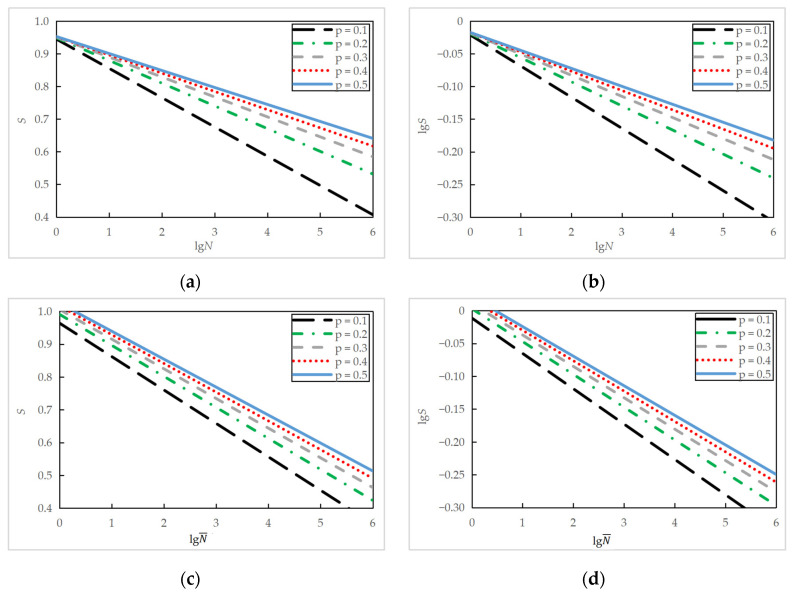
Fatigue *S*-*N* equations of PC series under different failure probabilities: (**a**) semi-logarithmic equation at a fixed minimum stress level of 0.10; (**b**) logarithmic equation at a fixed minimum stress level of 0.10; (**c**) semi-logarithmic equation when considering stress ratio; (**d**) logarithmic equation when considering stress ratio.

The fatigue equations of the plain concrete specimens under different failure probabilities are also shown in Figure 16. It is demonstrated in the figure that the lower the failure probability, the shorter the fatigue life at a given stress level. Similarly, semi-logarithmic and logarithmic equations were identical for the stress levels most likely encountered in practical applications. When considering the stress ratio, the slope coefficients are more stable, and the intercept coefficients are more evenly distributed. Probabilistic *S*-*N* curves have similar slopes, but the intercept increases with higher failure probability [9].

#### 3.2.4. Comparison between Fatigue Equations

In this section, we compare the fatigue equations obtained from the experimental investigation with those proposed by other scholars based on their fatigue test results. Since probabilistic fatigue equations are not available explicitly in the literature, only the mean fatigue equations were analyzed. Similarly, the comparison focuses on the fatigue analysis results of the plain concrete specimens.

**Figure 17:**
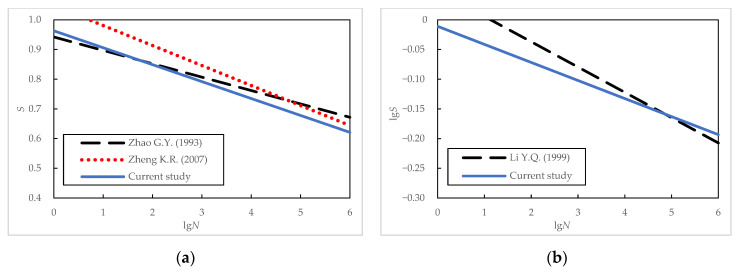
Comparison of fatigue equations at a fixed minimum stress level of 0.10: (**a**) semi-logarithmic equations; (**b**) logarithmic equations [7,8,13].

When the minimum stress level was fixed at 0.10, the fatigue equations of the plain concrete specimens obtained in the current study were compared with those proposed in References [7,8,13]. A summary of the various fatigue test programs along with the obtained fatigue equations are shown in Table 13, and a graphic representation of the equations is shown in Figure 17. It should be noted that the test condition in Reference [8] was three-point bending.

As can be seen in Figure 17, the fatigue equation proposed in the current work is relatively close to those proposed in the literature. For most fatigue problems in bridge engineering, the fatigue life of concrete usually lies within the range of 100~100,000 cycles, and the maximum stress levels are between 0.70 and 0.85. Compared to the high-strength concrete in References [7,8,13], the normal-grade C50 concrete in this study had a lower static flexural strength *f* and slightly lower fatigue strength. The large discreteness of the test results observed in the test program might have caused some discrepancies. The difference in the *S*-*N* curves’ slope in Figure 17b was probably caused by the difference in the test set-up. Under three-point bending, shear force exists at the cross-section where the beam specimen bears the maximum bending moment. The combined effect of the shear force and the bending moment causes more severe conditions, thus causing the slope of the fatigue equation proposed in Reference [8] to be steeper.

**Figure 18:**
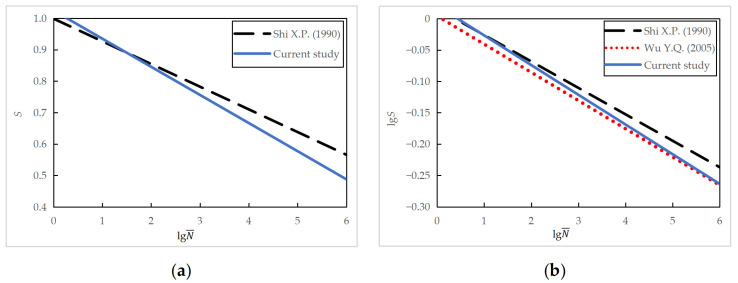
Comparison of fatigue equations considering the effect of stress ratio: (**a**) semi-logarithmic equations; (**b**) logarithmic equations [9,10].

When the effect of the stress ratio *R* is considered, the fatigue equations of the plain concrete specimens obtained in the current study were compared with those proposed in References [9,10]. A summary of various fatigue test programs and the obtained fatigue equations are shown in Table 14. A graphic representation of these equations is shown in Figure 18.

It can be seen in Table 14 and Figure 18 that the fatigue equations from three sources had slight differences in their slope and intercept coefficients. The fatigue equation proposed in the current work lies between those in References [9,10] for the range of stress levels and fatigue life of practical concern. Note that the static flexural strength *f* of three batches of concrete was relatively close. In short, the difference between the fatigue equations obtained from similar test conditions when considering the effect of the stress ratio is slight. Regarding the influencing factors for the fatigue characteristics of concrete, both the maximum stress level and stress ratio are essential parameters, followed by the influence of the concrete strength grade, with slightly less significance. This observation confirms the conclusions from earlier research [28].

### 3.3. Future Research

Large discreteness in the fatigue life and longitudinal strain of concrete was observed for specimens tested under the same stress conditions, even in carefully controlled fatigue tests. This large discreteness was partly caused by imperfections in the fatigue testing machine and measuring techniques. It also reflects that concrete fatigue is a complicated problem, and deep understanding of the important affecting parameters and the failure mechanism still needs to be improved. With upgraded test facilities, more fatigue tests with lower stress levels (0.65~0.75) and possibly extending the fatigue loading up to 2 million cycles are desirable in future investigations.

The longitudinal strain in nearly 100 concrete specimens was continuously recorded during the fatigue test. The maximum strain just before fatigue failure was analyzed. Much work is required to utilize the collected strain data fully. For example, the evolution of the maximum, minimum, residual strains, and strain amplitude during the entire process of fatigue loading is still ongoing, from which a strain-based damage variable could be constructed, and its evolution studied. Following this, the evolution of the elasticity modulus of concrete (both tangent and secant modulus) should be analyzed. Eventually, a cyclic constitutive model will be proposed, considering flexural tensile fatigue damage. With the aid of standard finite element software, the fatigue behavior of structural components such as concrete bridge decks, could be practically predicted with relative ease.

At last, it should be pointed out that only the Chinese standard was used in this study. One might justify that concrete is an engineering product highly dependent on local raw materials, and concrete must be produced, tested, and evaluated strictly according to national standards. Differences in the technical rules exist between various national and international codes and standards. For example, while Φ150 mm × 300 mm cylinders are adopted to obtain the axial compressive strength in CEB-FIP, 150 mm × 150 mm × 300 mm prisms are specified in the governing Chinese code [25,29]. It would be desirable to compare CEB-FIP, Eurocode, RILEM, ACI, and the Chinese codes and standards regarding the requirements for mix design, slump evaluation, fatigue test specimens, and recommended *S*-*N* curves. Comparative studies, like the one in the literature [30], should be conducted through international cooperation. This effort could contribute to avoiding repetition and confusion within the academic community and encouraging the convergence and standardization of sustainable development.

## 4. Conclusions

Four-point bending fatigue tests of 55 plain concrete (PC series) and 42 reinforced concrete (RC series) beams were conducted for the flexural fatigue properties of normal-grade C50 ordinary concrete, and the fatigue life data of test specimens were analyzed and fitted to semi-logarithmic and logarithmic equations. Probabilistic fatigue equations with a failure probability *p* varying from 0.1 to 0.5 for specimens with a fixed minimum stress level of 0.10, and when considering a stress ratio *R* varying from about 0.11 to 0.44, were obtained respectively through regression analysis. These fatigue equations were then compared to those available in the literature. The following conclusions are drawn:

(1) Two-parameter Weibull distribution could describe the fatigue life of specimens tested under the same maximum stress level *S*_max_. The semi-logarithmic and logarithmic equations were nearly identical at the tested stress levels, with the latter predicting longer fatigue life for *S*_max_ < 0.70.
(2) The stress ratio *R* is an essential factor affecting the fatigue life of concrete, the effect of which shall not be ignored and can be conveniently considered through the equivalent fatigue life $\bar{N}=N^{1-R}$.
(3) Although the PC specimens failed in brittle fracture and the RC series exhibited ductile behavior after macroscopic concrete cracking appeared, the fatigue cracking life of these series are relatively close, with the latter slightly longer. The restraining effect from steel reinforcement influences the fatigue crack initiation of concrete.
(4) Fatigue equations of normal-grade C50 ordinary concrete lie slightly below those of high-strength concrete. Although not as important as the maximum stress level and the stress ratio, the material strength grade affects ordinary concrete’s flexural fatigue properties.
(5) During the stress-controlled bending fatigue tests, the measured concrete strain developed in a three-stage manner with a continuously increasing value. The maximum longitudinal strain in the concrete just before fatigue failure was in reverse proportion to the maximum stress level applied.

In the future, the evolution of cyclic strain, the stiffness degradation, and the fatigue damage constitutive relationship shall be established to lay a foundation for the fatigue assessment of reinforced concrete structures.

## Figures and Tables

**Table 1:** Details of concrete mix.

Materials	Cement	Fine Aggregate	Coarse Aggregate	Water	Admixture	Mineral Powder	Fly Ash
**Properties**	P.O 42.5	Medium sand	Crushed, 5~25 mm	–	STD-PCS	S95	IIF
**Amount (kg/m^3^)**	347	670	1048	160	9.2	69	46
**Mix proportion**	1	1.93	3.02	0.46	0.03	0.20	0.13

**Table 2:** Fatigue test matrix of PC series (plain concrete specimens) and RC series (reinforced concrete specimens).

Series	Set No.	Maximum Stress Level *S*_max_	Minimum Stress Level *S*_min_	Stress Ratio *R*	Number of Specimens	Test Frequency (Hz)
PC	1	0.90	0.10	0.110	5	0.1
	2	0.90	0.25	0.280	5	0.1
	3	0.90	0.40	0.440	5	0.1
	4	0.80	0.10	0.125	12	5
	5	0.75	0.10	0.133	13	5
	6	0.75	0.25	0.333	12	5
	7	0.65	0.10	0.154	3	5
RC	1	0.85	0.10	0.117	7	5
	2	0.85	0.25	0.294	7	5
	3	0.80	0.10	0.125	7	5
	4	0.80	0.25	0.313	7	5
	5	0.75	0.10	0.133	7	5
	6	0.75	0.25	0.333	7	5

**Table 3:** Compressive strength of concrete mixes.

Series	Measured Compressive Strength (MPa)						Statistical Characteristics		
	Cube 1	Cube 2	Cube 3	Cube 4	Cube 5	Cube 6	Mean Value (MPa)	Standard Deviation (MPa)	Coefficient of Variation
PC	51.0	47.4	61.2	41.6	61.6	60.8	53.9	7.77	0.14
RC	51.2	52.1	51.4	50.2	51.5	50.2	51.1	0.76	0.015

**Table 4:** Static flexural strength of concrete mixes.

Series	Measured Flexural Strength (MPa)								Statistical Characteristics		
	Set 1	Set 2	Set 3	Set 4	Set 5	Set 6	Set 7	Set 8	Mean Value (MPa)	Standard Deviation (MPa)	Coefficient of Variation
PC	6.6	5.3	6.0	–	–	–	–	–	5.6	0.55	0.10
	5.9	4.8	5.4	–	–	–	–	–			
	6.1	5.2	5.4	–	–	–	–	–			
RC	5.1	5.3	5.1	4.3	4.6	4.7	5.6	4.7	5.1	0.44	0.09
	5.2	3.8	4.5	4.8	4.2	4.5	5.6	5.3			
	4.5	4.7	4.9	4.7	3.8	4.8	5.5	5.4			

**Table 5:** Fatigue life test results of plain concrete specimens (PC series).

Set No.	Set Designation	*S*_max_	*S*_min_	Specimen Designation	Fatigue Life *N* (Cycle)	Specimen Failure Order	Fatigue Life *N* (Cycle)	Failure Probability *p*
1	S-90-1	0.90	0.10	S-90-1-1	12	1	3	0.17
				S-90-1-2	9	2	8	0.33
				S-90-1-3	9	3	9	0.50
				S-90-1-4	3	4	9	0.67
				S-90-1-5	8	5	12	0.83
2	S-90-25	0.90	0.25	S-90-25-1	415	1	110	0.17
				S-90-25-2	216	2	196	0.33
				S-90-25-3	110	3	216	0.50
				S-90-25-4	196	4	234	0.67
				S-90-25-5	234	5	415	0.83
3	S-90-4	0.90	0.40	S-90-4-1	1347	1	136	0.17
				S-90-4-2	136	2	370	0.33
				S-90-4-3	934	3	620	0.50
				S-90-4-4	620	4	934	0.67
				S-90-4-5	370	5	1347	0.83
4	S-80-1	0.80	0.10	S-80-1-1	3872	1	96	0.08
				S-80-1-2	96	2	261	0.17
				S-80-1-3	496	3	496	0.25
				S-80-1-4	6115	4	618	0.33
				S-80-1-5	1391	5	1036	0.42
				S-80-1-6	261	6	1391	0.50
				S-80-1-7	6358	7	3872	0.58
				S-80-1-8	61,960 ^a^	8	6115	0.67
				S-80-1-9	1036	9	6358	0.75
				S-80-1-10	618	10	6383	0.83
				S-80-1-11	8930	11	8930	0.92
				S-80-1-12	6383	12	61,960 ^a^	–
5	S-75-1	0.75	0.10	S-75-1-1	1658	1	63	0.08
				S-75-1-2	32,835	2	304	0.15
				S-75-1-3	14,444	3	380	0.23
				S-75-1-4	7574	4	528	0.31
				S-75-1-5	304	5	1658	0.38
				S-75-1-6	380	6	7574	0.46
				S-75-1-7	528	7	11,328	0.54
				S-75-1-8	63	8	14,048	0.62
				S-75-1-9	14,048	9	14,444	0.69
				S-75-1-10	224,931 ^a^	10	19,585	0.77
				S-75-1-11	11,328	11	32,835	0.85
				S-75-1-12	49,629	12	49,629	0.92
				S-75-1-13	19,585	13	224,931 ^a^	–
6	S-75-25	0.75	0.25	S-75-25-1	54,815	1	2518	0.08
				S-75-25-2	2518	2	5321	0.17
				S-75-25-3	365,759 ^a^	3	7308	0.25
				S-75-25-4	34,187	4	14,209	0.33
				S-75-25-5	5321	5	21,097	0.42
				S-75-25-6	7308	6	26,049	0.50
				S-75-25-7	68,135	7	32,646	0.58
				S-75-25-8	32,646	8	34,187	0.67
				S-75-25-9	26,049	9	39,379	0.75
				S-75-25-10	39,379	10	54,815	0.83
				S-75-25-11	14,209	11	68,135	0.92
				S-75-25-12	21,097	12	365,759 ^a^	–
7	S-65-1	0.65	0.10	S-65-1-1	1,000,000 *	1	1,000,000 *	–
				S-65-1-2	1,000,000 *	2	1,000,000 *	–
				S-65-1-3	1,000,000 *	3	1,000,000 *	–

^a^ Rejected as an outlier by Chauvenet’s criterion, not included in further analysis. * Treated as run-out specimens, not included in further analysis.

**Table 6:** Fatigue cracking life test results of reinforced concrete specimens (RC series).

Set Designation	J-S-85-1	J-S-85-25	J-S-80-1	J-S-80-25	J-S-75-1	J-S-75-25
*S*_max_	0.85	0.85	0.80	0.80	0.75	0.75
*S*_min_	0.10	0.25	0.10	0.25	0.10	0.25
**Specimen designation** **Fatigue cracking life *N*** **(cycle)**	J-S-85-1-2	J-S-85-25-2	J-S-80-1-1	J-S-80-25-4	J-S-75-1-1	J-S-75-25-1
	228	1765	3782	4870	13,213	598 ^a^
	J-S-85-1-3	J-S-85-25-5	J-S-80-1-6	J-S-80-25-7	J-S-75-1-3	J-S-75-25-2
	516	1976	4028	6780	14,274	32,730
	J-S-85-1-5	J-S-85-25-4	J-S-80-1-3	J-S-80-25-6	J-S-75-1-4	J-S-75-25-3
	586	2249	4517	8976	14,680	35,214
	J-S-85-1-6	J-S-85-25-1	J-S-80-1-2	J-S-80-25-5	J-S-75-1-2	J-S-75-25-5
	611	2331	5087	9307	16,030	38,683
	J-S-85-1-4	J-S-85-25-3	J-S-80-1-4	J-S-80-25-1	J-S-75-1-7	J-S-75-25-6
	634	2544	5226	9696	23,793	41,878
	J-S-85-1-7	J-S-85-25-6	J-S-80-1-7	J-S-80-25-2	J-S-75-1-5	J-S-75-25-4
	691	4221	5459	9900	38,588	41,908
	J-S-85-1-1	J-S-85-25-7	J-S-80-1-5	J-S-80-25-3	J-S-75-1-6	J-S-75-25-7
	1800	6185	5857	10,954	72,320	48,434

^a^ Rejected as an outlier by Chauvenet’s criterion, not included in further analysis.

**Table 7:** Measured strains in plain concrete specimens (PC series) at the end of the fatigue test.

Specimen Designation	Maximum Strain (με)	Minimum Strain (με)	Residual Strain (με)	Deformation Modulus (MPa)
S-90-1-1	789	278	219	9783
S-90-1-5	367	111	84	19,531
Mean value	578	194	152	14,657
C.V.	0.52	0.61	0.63	0.47
S-90-25-1	456	222	125	16,439
S-90-25-2	1000	522	314	7788
S-90-25-3	1167	622	395	7028
S-90-25-5	411	289	218	23,271
Mean value	758	478	309	12,696
C.V.	0.50	0.36	0.29	0.72
S-90-4-1	656	467	312	11,456
S-90-4-2	967	511	212	5968
S-90-4-3	989	678	418	8114
S-90-4-4	556	333	141	11,912
S-90-4-5	389	233	104	19,263
Mean value	711	444	237	11,343
C.V.	0.37	0.38	0.54	0.45
S-80-1-1	1278	767	678	8002
S-80-1-3	733	422	363	12,723
S-80-1-4	1344	533	383	3617
S-80-1-5	856	311	210	7010
S-80-1-7	256	122	97	29,873
S-80-1-9	1033	578	484	8176
S-80-1-10	1011	433	312	6361
S-80-1-11	367	200	168	25,696
S-80-1-12	444	267	232	21,218
Mean value	814	404	325	13,631
C.V.	0.48	0.50	0.55	0.70
S-75-1-2	267	156	133	33,763
S-75-1-7	1156	500	377	5786
S-75-1-8	733	267	179	8125
S-75-1-9	989	533	444	8255
S-75-1-11	1878	722	534	3101
S-75-1-12	1511	944	834	6676
S-75-1-13	1144	656	559	6656
Mean value	1097	423	344	12,082
C.V.	0.47	0.66	0.69	0.87
S-75-25-2	1222	744	467	5957
S-75-25-4	1456	922	627	4876
S-75-25-5	1200	811	588	7330
S-75-25-6	622	422	306	14,117
S-75-25-7	411	244	143	18,599
S-75-25-8	1444	933	676	4975
S-75-25-9	1741	1022	528	2737
S-75-25-10	1100	778	581	9238
S-75-25-11	1367	856	559	5118
S-75-25-12	1078	667	437	6135
Mean value	1164	740	491	7908
C.V.	0.34	0.33	0.33	0.62

Note: C.V. stands for coefficient of variation.

**Table 8:** Measured strains in reinforced concrete specimens (RC series) at fatigue cracking initiation.

Specimen Designation	Maximum Strain (με)	Minimum Strain (με)	Residual Strain (με)	Deformation Modulus (MPa)
J-S-85-1-1	562	281	226	12,867
J-S-85-1-2	610	274	219	11,362
J-S-85-1-3	575	212	145	10,321
J-S-85-1-4	486	185	125	12,299
J-S-85-1-5	767	397	331	10,202
J-S-85-1-6	788	274	177	7268
J-S-85-1-7	664	295	226	10,166
Mean value	636	274	207	10,641
C.V.	0.17	0.25	0.33	0.17
J-S-85-25-1	726	432	295	10,322
J-S-85-25-2	685	432	314	11,996
J-S-85-25-3	801	493	350	9863
J-S-85-25-4	685	418	293	11,430
J-S-85-25-5	658	418	309	12,848
J-S-85-25-6	466	260	168	15,030
J-S-85-25-7	644	445	309	13,484
Mean value	666	414	291	12,139
C.V.	0.15	0.18	0.20	0.15
J-S-80-1-1	432	130	78	11,875
J-S-80-1-2	541	233	175	11,467
J-S-80-1-3	925	493	410	8153
J-S-80-1-4	870	479	404	9013
J-S-80-1-5	589	295	244	12,201
J-S-80-1-6	897	575	388	8638
J-S-80-1-7	568	336	294	15,204
Mean value	689	363	285	10,936
C.V.	0.29	0.44	0.45	0.23
J-S-80-25-1	555	329	219	12,601
J-S-80-25-2	1055	753	598	9142
J-S-80-25-3	692	452	332	11,689
J-S-80-25-5	658	452	337	12,873
J-S-80-25-6	740	514	386	11,718
J-S-80-25-7	411	281	206	20,139
Mean value	685	463	346	13,027
C.V.	0.31	0.36	0.41	0.29
J-S-75-1-1	808	507	448	10,942
J-S-75-1-2	740	418	368	10,775
J-S-75-1-3	973	651	601	10,780
J-S-75-1-4	712	384	334	10,582
J-S-75-1-5	315	116	78	16,540
J-S-75-1-6	733	452	409	12,364
J-S-75-1-7	630	438	409	18,078
Mean value	702	424	378	12,866
C.V.	0.29	0.38	0.42	0.24
J-S-75-25-3	486	322	240	16,237
J-S-75-25-4	603	418	325	14,428
J-S-75-25-5	747	500	377	10,826
J-S-75-25-6	849	610	493	11,286
J-S-75-25-7	452	281	195	15,588
Mean value	627	426	326	13,673
C.V.	0.27	0.31	0.36	0.18

Note: C.V. stands for coefficient of variation.

**Table 9:** Regression analysis results of Weibull distribution test.

Minimum Stress Level	Series	Maximum Stress Level	Number of Specimens	Regression Coefficient *m*	Regression Coefficient ln*t*_0_	Coefficient of Determination *R*^2^
Fixed at 0.10	PC	0.90	5	1.5188	3.5137	0.83
		0.80	11	0.6571	5.3104	0.96
		0.75	12	0.4607	4.2646	0.95
	RC	0.85	7	1.2703	8.6111	0.77
		0.80	7	5.3293	45.6245	0.98
		0.75	7	1.2339	12.8204	0.81
Varying (stress ratio considered)	PC	0.90	15	1.0060	3.5847	0.95
		0.80	11	0.7509	5.3104	0.96
		0.75	23	0.7577	5.7302	0.98
	RC	0.85	14	2.2903	13.4023	0.86
		0.80	14	1.5054	10.7672	0.86
		0.75	13	1.0038	8.4708	0.82

**Table 10:** Summary of mean fatigue *S*-*N* equations.

Series	Analyzed Specimens	Semi-Logarithmic Equations	Logarithmic Equations
PC	Constant *S*_min_ = 0.10	S=0.9631−0.0570 lgN	lgS=−0.0109−0.0304 lgN
	Varying *S*_min_, R considered	S=1.0247−0.0895(1−R) lgN	lgS=0.0208−0.0474(1−R) lgN
RC	Constant *S*_min_ = 0.10	S=1.0307−0.0639 lgN	lgS=0.0281−0.0348 lgN
	Varying *S*_min_, R considered	S=1.0946−0.0999(1−R) lgN	lgS=0.0627−0.0543(1−R) lgN

**Table 11:** Fatigue life (and equivalent fatigue life $\bar{N}$ when considering *R*) in cycles at various failure probabilities.

Analyzed Specimens	Failure Probability *p*	PC Series Maximum Stress Level			RC Series Maximum Stress Level		
		0.90	0.80	0.75	0.85	0.80	0.75
Constant *S*_min_ = 0.10	0.1	2	105	79	149	3425	5252
	0.2	4	330	404	270	3943	9648
	0.3	5	674	1118	390	4305	14,110
	0.4	6	1164	2437	518	4606	18,878
	0.5	8	1852	4728	659	4877	24,176
Varying *S*_min_, *R* considered	0.1	4	59	99	130	286	491
	0.2	8	160	266	181	472	1037
	0.3	13	299	494	222	644	1655
	0.4	18	482	793	259	817	2367
	0.5	25	723	1187	296	1001	3209

**Table 12:** Coefficients from regression analysis of probabilistic fatigue equations.

Analyzed Specimens	Series	Failure Probability *p*	Semi-Logarithmic Equations			Logarithmic Equations		
			Intercept Coefficient *a*	Slope Coefficient *b*	Coefficient of Determination *R*^2^	Intercept Coefficient *A*	Slope Coefficient *B*	Coefficient of Determination *R*^2^
Constant *S*_min_ = 0.10	PC	0.1	0.9446	0.0896	0.85	−0.0213	0.0476	0.83
		0.2	0.9488	0.0696	0.92	−0.0192	0.0368	0.90
		0.3	0.9506	0.0610	0.94	−0.0183	0.0323	0.93
		0.4	0.9517	0.0558	0.95	−0.0178	0.0295	0.94
		0.5	0.9525	0.0520	0.96	−0.0174	0.0275	0.95
	RC	0.1	1.0034	0.0647	0.84	0.0140	0.0355	0.83
		0.2	1.0148	0.0644	0.92	0.0200	0.0352	0.91
		0.3	1.0220	0.0642	0.96	0.0237	0.0350	0.96
		0.4	1.0274	0.0640	0.98	0.0265	0.0349	0.98
		0.5	1.0320	0.0639	1.00	0.0288	0.0348	0.99
Varying *S*_min_, *R* considered	PC	0.1	0.9641	0.1019	0.97	−0.0113	0.0538	0.96
		0.2	0.9909	0.0946	0.96	0.0029	0.0500	0.95
		0.3	1.0058	0.0905	0.96	0.0107	0.0478	0.95
		0.4	1.0163	0.0876	0.96	0.0163	0.0463	0.95
		0.5	1.0248	0.0853	0.95	0.0208	0.0451	0.94
	RC	0.1	1.2199	0.1734	0.99	0.1313	0.0945	0.98
		0.2	1.1490	0.1318	1.00	0.0925	0.0717	0.99
		0.3	1.1197	0.1146	1.00	0.0765	0.0623	1.00
		0.4	1.1020	0.1041	1.00	0.0668	0.0566	1.00
		0.5	1.0893	0.0967	1.00	0.0599	0.0526	1.00

**Table 13:** Comparison of fatigue equations obtained for a fixed minimum stress level of 0.10.

Reference	*f* (MPa)	Specimen Dimension (cm)	Number of Datapoints	*S*_min_	*S*_max_	Test Condition	Test Frequency (Hz)	Fatigue Equations
Zhao G.Y. (1993) [7]	7.43	10 × 10 × 40	16	0.10	0.70~0.90	Four-point bending	5~10	S=0.942−0.045 lgN
Li Y.Q. (1999) [8]	7.68	10 × 10 × 51.5	60	0.10	0.60~0.90	Three-point bending	10	lgS=0.0483−0.0426 lgN
Zheng K.R. (2007) [13]	7.6	10 × 10 × 40	57	0.10	0.65~0.90	Four-point bending	2~10	S=1.04808−0.0673 lgN
Current study	5.6	15 × 15 × 55	28	0.10	0.75~0.90	Four-point bending	0.1~5	S=0.9631−0.0570 lgN lgS=−0.0109−0.0304 lgN

**Table 14:** Comparison of fatigue equations considering the effect of stress ratio *R*.

Reference	*f* (MPa)	Specimen Dimension (cm)	Number of Datapoints	*R*	*S*_max_	Test Condition	Test Frequency (Hz)	Fatigue Equations
Shi X.P. (1990) [9]	6.08	10 × 10 × 50	73	0.08~0.5	0.55~0.90	Four-point bending	1~20	S=0.999−0.0722(1−R) lgN lgS=0.0162−0.0422(1−R) lgN
Wu Y.Q. (2005) [10]	5.1	10 × 10 × 40	84	0.1~0.5	0.625~0.9	Four-point bending	1~20	lgS=0.0044−0.045(1−R) lgN
Current study	5.6	15 × 15 × 55	49	0.11~0.44	0.75~0.90	Four-point bending	0.1~5	S=1.0247−0.0895(1−R) lgN lgS=0.0208−0.0474(1−R) lgN

## Data Availability

Details of the experimental data presented in this study are available upon reasonable request to the corresponding author.
